# Human Infections with *Plasmodium knowlesi,* the Philippines

**DOI:** 10.3201/eid1405.071407

**Published:** 2008-05

**Authors:** Jennifer Luchavez, Fe Espino, Peter Curameng, Ronald Espina, David Bell, Peter Chiodini, Debbie Nolder, Colin Sutherland, Kim-Sung Lee, Balbir Singh

**Affiliations:** * Research Institute for Tropical Medicine, Muntinlupa City, the Philippines; † Center for Health Development, Puerto Princesa City, the Philippines; ‡ World Health Organization Regional Office for the Western Pacific, Manila, the Philippines; § London School of Hygiene & Tropical Medicine, London, UK; ¶ Universiti Malaysia Sarawak, Sarawak, Malaysia

**Keywords:** Malaria, *Plasmodium knowlesi*, Philippines, zoonoses, dispatch

## Abstract

Five human cases of infection with the simian malaria parasite *Plasmodium knowlesi* from Palawan, the Philippines, were confirmed by nested PCR. This study suggests that this zoonotic infection is found across a relatively wide area in Palawan and documents autochthonous cases in the country.

## Abstract 2

Human Infections with *Plasmodium knowlesi*, the Philippines

Human malaria is commonly caused by *Plasmodium falciparum, P. vivax, P. malariae,* and *P. ovale*. However, a large focus of human infections with the simian malaria parasite, *P. knowlesi* (*1*)*,* has recently been reported in Malaysian Borneo (*2*), and single case reports of infections acquired in Thailand (*3*) and Myanmar (*4*) have been documented. The diagnosis of *P. knowlesi* in humans may be missed by microscopy since the early blood stages of *P. knowlesi* morphologically resemble *P. falciparum*; the mature blood stages and gametocytes are similar to those of *P. malariae* (*2*)*.*

## The Study

Palawan is an island province lying southwest of the main islands of the Philippines. One of its smaller islands, Balabac, located off the southern tip, is separated from Borneo by the Balabac Strait (Figure). Malaria transmission occurs in all 19 municipalities of the province throughout the year. The *Anopheles flavirostris* mosquito is the reported primary vector in the area (*5*). Based on national control program data in 2005, a total of 16,339 malaria cases were reported from Palawan, accounting for 35% of the country’s total. Of these, 11,580 (≈71%) were *P. falciparum*, 4,194 (26%) were *P. vivax*, 430 (3%) were *P. malariae*, and the remainder (135, <1%) were mixed-species infections.

**Figure:**
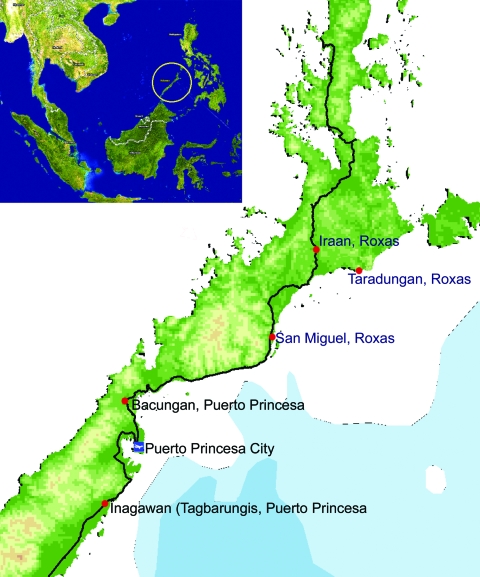
Map of Palawan, the Philippines, showing areas (red dots) where human *Plasmodium knowlesi* infections were confirmed (obtained from ESRI ArcGis 9 Media Kit and Provincial Development Office, Palawan, the Philippines).

Blood films of 2 patients (A and B), whose condition was diagnosed by microscopy as *P. malariae* at a local laboratory in Palawan, were sent to the malaria national reference laboratory of the Research Institute for Tropical Medicine (RITM) in Manila. Microscopy showed mature trophozoites indistinguishable from *P. malariae* and young ring forms of *P. falciparum.* This observation, and the fact that macaques in the Philippines can harbor *P. knowlesi* (*6**,**7*), raised the possibility that these 2 patients were infected with *P. knowlesi*. Therefore, replicate slides were sent to the Malaria Research Centre, University of Malaysia Sarawak (UNIMAS), for confirmation of the identity of the *Plasmodium* species by molecular methods. DNA was extracted from the 2 slides **as detailed previously (*8*)** and examined by nested PCR assays for *P. falciparum, P. vivax, P. malariae, P. knowlesi*, and *P. ovale* as described by Singh and co-workers (*2*). One sample was positive for *P. knowlesi* mono-infection; the other was a mixed infection of *P. falciparum, P. malariae*, and *P. knowlesi*. PCR results were confirmed on subsequent testing at the malaria reference laboratory, London School of Hygiene & Tropical Medicine, United Kingdom.

The blood films had been collected in January 2006 from 2 men (>40 years of age) who lived in the villages of Tagbarungis and Bacungan near Puerto Princesa City, Palawan (Figure). The patients were interviewed in July 2006 after their blood films were found to contain *P. knowlesi.* Both were subsistence farmers who had engaged in livelihood activities at night (charcoal making) in forested areas within a 50-km radius from their homes; neither had traveled out of Palawan within the past year. Before seeking medical treatment at the Palawan provincial malaria laboratory, they experienced chills, minor headaches, and daily low-grade fever, consistent with the reported quotidian fever pattern of this infection (*1*). *P. malariae* infection was diagnosed in both men by the local microscopist; each claimed to have responded well to chloroquine and primaquine drug therapy. The usual treatment regimen for *P. falciparum*/*P. malariae* was followed: 4 tablets of chloroquine (150 mg base/tablet) on days 1 and 2, and 2 tablets on day 3; 3 tablets of primaquine (15 mg/tablet) on day 4). The farmers reportedly stayed overnight before onset of their illness in forested foothills that contained many breeding sites ideal for *A. flavirostris* mosquitoes. Long-tailed macaques (*Macaca fascicularis*), the natural hosts for *P. knowlesi*, were observed to be roaming freely in the area. An additional 9 samples (D,E, G, H, I, J, K, O, and P), consisting of 5 blood films and 4 blood spots on filter paper, were obtained from patients at Bataraza and Roxas municipalities (also in Palawan) and *P. malariae* infection was diagnosed by the local microscopists. These samples were subsequently examined by nested PCR assays at UNIMAS after DNA extraction. Three were identified as *P. knowlesi*, 4 as *P. malariae*, and the remaining 2 as mixed species infections (Table). The findings of autochthonous *P. malariae* infections further compounded the problem of accurate diagnosis of *P. knowlesi* by microscopy. The *P. knowlesi* patients came from 3 different villages in Roxas, 80–100 km north of where the original 2 *P. knowlesi* case-patients resided, near Puerto Princesa (Figure). This suggests that human *P. knowlesi* infections are found across a relatively wide area in Palawan. PCR examination of more blood samples in other areas where *P. malariae* infections have been diagnosed by microscopy are necessary to determine the geographic distribution and public health importance of human knowlesi infections in the Philippines.

**Table:** Microscopy and PCR results of blood samples from Palawan, the Philippines

Patient	Age, y/sex	Location	*Plasmodium* species	
			Microscopy	PCR
A	50/M	Bacungan, Puerto Princesa	*P. falciparum* (gametocytes), *P. malariae*	*P. falciparum, P. malariae, P. knowlesi*
B	49/M	Inagawan, Tagbarungis, Puerto Princesa	*P. falciparum, P. malariae*	*P. knowlesi*
D	55/F	Caibulo, Iraan, Roxas	*P.malariae*	*P. knowlesi*
E	3/M	Balogo, San Miguel, Roxas	*P.malariae*	*P. knowlesi*
G	6/M	Maninguin, Iraan, Roxas	*P. malariae*	*P. malariae*
H	25/M	Minara, Roxas	*P. malariae*	*P. malariae*
I	10/F	Taradungan, Roxas	*P. malariae*	*P. knowlesi*
J	5/M	Bono-Bono, Bataraza	*P. vivax, P. malariae*	*P. falciparum, P. vivax, P. malariae*
K	14/F	Bono-Bono, Bataraza	*P. malariae*	*P. malariae*
O	9/M	Inogbong,Bataraza	*P. malariae*	*P. malariae*
P	5/F	Inogbong, Bataraza	*P. falciparum, P. malariae*	*P. falciparum, P. vivax*

## Conclusions

This report extends the geographic range of human *P. knowlesi* infections from Thailand (*3*), Myanmar (*4*), peninsular Malaysia (*8*), and Malaysian Borneo (*2*) to Palawan Island in the Philippines. Although the parasite has been isolated from local macaques in the Philippines in 1961 (*6*) and 1978 (*7*), this report documents autochthonous human cases in the country. Major progress in malaria control has been achieved in many malarious areas in the Philippines (*9*). However, *P. knowlesi* forms a previously unrecognized pool of infections that may be maintained in forested areas through its presence in a simian reservoir, despite control efforts in the human population. Current data suggest that human knowlesi malaria is strictly a zoonotic disease. To confirm this theory, further knowledge of the dynamics of human infection is needed.

## References

[1] Garnham PCC. Malaria parasites and other haemosporidia. Oxford: Blackwell Scientific Publications; 1966. p. 317–32.

[2] Singh B, Kim Sung L, Matusop A, Radhakrishnan A, Shamsul SS, Cox-Singh J, A large focus of naturally acquired *Plasmodium knowlesi* infections in human beings. Lancet. 2004;363:1017–24. 10.1016/S0140-6736(04)15836-4 15051281

[3] Jongwutiwes S, Putaporntip C, Iwasaki T, Sata T, Kanbara H. Naturally acquired *Plasmodium knowlesi* malaria in humans, Thailand. Emerg Infect Dis. 2004;10:2211–3. 15663864 10.3201/eid1012.040293 PMC3323387

[4] Zhu HM, Li J, Zheng H. Human natural infection of *Plasmodium knowlesi.* Chinese Journal of Parasitology and Parasitic Diseases. 2006;24:70–1. 16866152

[5] Oberst R, Schultz G, Laughlin L, Sy N, Santos M, Casimiro C. Epidemiological study of malaria in Palawan. The Philippine Journal of Microbiology and Infectious Diseases. 1988;17:41–8 [cited 2008 Mar 8]. Available from http://www.psmid.org.ph/index.php?fid=journals/Volume17Number2

[6] Lambrecht FL, Dunn FL, Eyles DE. Isolation of *Plasmodium knowlesi* from Philippine macaques. Nature. 1961;191:1117–8. 10.1038/1911117a0 13758508

[7] Tsukamoto M, Miyata A, Miyagi I. Surveys on simian malaria parasites and their vectors in Palawan Island, the Philippines. Institute of Tropical Medicine, Nagasaki University, Japan. 1978;20:29–50 [cited 2008 Mar 8]. Available from http://naosite.lb.nagasaki-u.ac.jp/dspace/handle/10069/4234

[8] Cox-Singh J, Davis TME, Lee KS, Shamsul SSG, Divis PCS, Matusop A, *Plasmodium knowlesi* malaria in humans is widely distributed and potentially life threatening. Clin Infect Dis. 2008;46:165–71. 10.1086/524888 18171245 PMC2533694

[9] National Objective for Health. Philippines, 2005–2010 [cited 2008 Jan 7]. Available from www2.doh.gov.ph/noh2007/NOHWeb32/NOHperSubj/Chap4/Malaria.pdf

